# Identification of Transmembrane Protease Serine 2 and Forkhead Box A1 As the Potential Bisphenol A Responsive Genes in the Neonatal Male Rat Brain

**DOI:** 10.3389/fendo.2018.00139

**Published:** 2018-03-28

**Authors:** Takayoshi Ubuka, Shogo Moriya, Tomoko Soga, Ishwar Parhar

**Affiliations:** Jeffrey Cheah School of Medicine and Health Sciences, Brain Research Institute Monash Sunway, Monash University Malaysia, Bandar Sunway, Selangor, Malaysia

**Keywords:** transmembrane protease serine 2, forkhead box A1, androgen receptor, estrogen receptor, polymerase chain reaction array, gonadotropin-inhibitory hormone neurons

## Abstract

Perinatal exposure of Bisphenol A (BPA) to rodents modifies their behavior in later life. To understand how BPA modifies their neurodevelopmental process, we first searched for BPA responsive genes from androgen and estrogen receptor signaling target genes by polymerase chain reaction array in the neonatal male rat brain. We used a transgenic strain of Wistar rats carrying enhanced green fluorescent protein tagged to gonadotropin-inhibitory hormone (GnIH) promoter to investigate the possible interaction of BPA responsive genes and GnIH neurons. We found upregulation of transmembrane protease serine 2 (*Tmprss2*), an androgen receptor signaling target gene, and downregulation of Forkhead box A1 (*Foxa1*), an ER signaling target gene, in the medial amygdala of male rats that were subcutaneously administered with BPA from day 1 to 3. Tmprss2-immunoreactive (ir) cells were distributed in the olfactory bulb, cerebral cortex, hippocampus, amygdala, and hypothalamus in 3 days old but not in 1-month-old male rats. Density of Tmprss2-ir cells in the medial amygdala was increased by daily administration of BPA from day 1 to 3. Tmprss2 immunoreactivity was observed in 26.5% of GnIH neurons clustered from the ventral region of the ventromedial hypothalamic nucleus to the dorsal region of the arcuate nucleus of 3-day-old male rat hypothalamus. However, *Tmprss2* mRNA expression significantly decreased in the amygdala and hypothalamus of 1-month-old male rats. *Foxa1* mRNA expression was higher in the hypothalamus than the amygdala in 3 days old male rats. Intense Foxa1-ir cells were only found in the peduncular part of lateral hypothalamus of 3-day-old male rats. Density of Foxa1-ir cells in the hypothalamus was decreased by daily administration of BPA from day 1 to 3. *Foxa1* mRNA expression in the hypothalamus also significantly decreased at 1 month. These results suggest that BPA disturbs the neurodevelopmental process and behavior of rats later in their life by modifying *Tmprss2* and *Foxa1* expressions in the brain.

## Introduction

Bisphenol A (BPA) is an organic synthetic compound widely used to make polycarbonate plastics and epoxy resins utilized in reusable food and drink containers and inner lining of cans and bottles (1). Hydrolysis of the ester-bond linking BPA can occur at high temperature, and BPA can be leached out into food and beverages. Fetuses or young animals during their developmental stages are thought to be susceptible to BPA exposure than adults (2). Therefore, detectable level of BPA in 88% of human cord blood samples (3) generated social concerns about the effect of BPA on the fetuses.

Dodds and Lawson were the first to show the estrogenic property of BPA (4). However, the estrogenic effect of BPA is 10,000 times less potent than estradiol-17β shown by uterine vascular permeability assay in ovariectomized mice (5). Similar level of agonistic activity of BPA to estrogen receptor (ER) was shown in yeast expressing human estrogen or androgen receptor (AR) (6). On the other hand, BPA is a potent ligand for the non-classic membrane bound G protein-coupled receptor for estrogen (GPR30) (7, 8). BPA did not have an agonistic activity but had an antagonistic activity to AR in the same yeast-based assay (6). Stronger antagonistic activity of BPA to human AR was shown in an African monkey kidney cell line (9). It is also known that BPA can disturb steroidogenesis by interfering the activity of steroidogenic enzymes, such as CYP450scc, 3βHSD, and CYP450arom (10). Perera et al. (11) examined the association between prenatal BPA exposure and child behavior. They found that high prenatal BPA exposure was associated with higher emotionally reactive and aggressive behavior syndromes in boys and lower anxious/depressed and aggressive behavior in girls (11). There are numerous studies showing the effect of perinatally administered BPA on social behavior of rodents. BPA exposure during pregnancy increased display of nose-to-nose contacts, play solicitations, and approaches in both sexes (12). Administration of BPA during pregnancy or lactation increased defensive behavior in male and sexual behavior in female rats (13). In the other study, BPA exposed males during gestation and lactation showed persistent deficits in sexual behavior in adulthood (14).

To find BPA responsive genes in the brain mediating its effect on social behavior, we first focused on amygdala, because amygdala is the integrative center for the processing of emotion, which is pivotal for social behavior. It is thought that deficits in the development of amygdala may cause autism, a neurodevelopmental disorder that is characterized by impaired social interaction (the amygdala theory of autism) (15). It is also known that these brain nuclei express ARs and ERs and abnormally enlarge in autism infants (16). We subcutaneously administered a reference dose of BPA (50 µg/kg BW/day) determined by United States Environmental Protection Agency (EPA, www.epa.gov/iris/subst/0356.htm) to neonatal male transgenic rats carrying enhanced green fluorescent protein tagged to gonadotropin-inhibitory hormone [EGFP-GnIH (17)] promoter from day 1 to 3 and differential expressions of AR and ER signaling target genes in the medial amygdala were analyzed by polymerase chain reaction (PCR) array. GnIH is a hypothalamic neuropeptide that decreases gonadotropin secretion directly acting on the pituitary or by decreasing the activity of gonadotropin-releasing hormone (GnRH) neurons (18, 19). Recent studies have shown that GnIH neurons also decrease motivated behavior (20, 21). We found upregulation of transmembrane protease serine 2 (*Tmprss2*) and downregulation of Forkhead box A1 (*Foxa1*) mRNAs in the amygdala by BPA exposure. We further studied the location of Tmprss2 and Foxa1 immunoreactive (ir) cells, effect of BPA on the density of Tmprss2 and Foxa1-ir cells and developmental changes in *Tmprss2* and *Foxa1* mRNA expressions in the male rat brain.

## Materials and Methods

### Animals

EGFP-GnIH Wistar rats (17) were housed under a controlled 12 h light/dark cycle (light on at noon) and the temperature was maintained at 22°C in a specific pathogen free animal facility. All rats had free access to autoclaved food and water. The pups were counted after parturition, weighed, and remained with their biological mother. All procedures were approved by Monash University, Animal Ethics Committee (MARP/2016/037).

### PCR Array

Bisphenol A at 50 µg/kg or vehicle (sesame oil) was subcutaneously injected to neonatal male rats after dawn daily from postnatal day 1 (P1) to P3. 2 h after the last injection the rats were deeply anesthetized by Zoletil/Ketamine/Xylazine (Z/K/X) at 13.5 mg/90 μl/kg and brains were collected and stored at −80°C. Medial amygdaloid tissue from both sides of the brain was dissected in a cryostat at −20°C by referring to a rat brain atlas (22). Medial amygdaloid tissues from two male siblings were combined in a tube for homogenization. Total medial amygdaloid tissue samples of six BPA treated rats from three different littermates and six vehicle treated rats from three different littermates were collected. Total RNA was extracted by using an RNA isolation kit (RNeasy mini kit; QIAGEN, Hilden, Germany) and reverse transcribed by using High-Capacity cDNA Reverse Transcription Kit (ThermoFisher SCIENTIFIC, Waltham, MA, USA). Differential gene expression was analyzed by PCR arrays, RT^2^ Profiler™ PCR Array Rat AR Signaling Targets, and RT^2^ Profiler™ PCR Array Rat ER Signaling Targets (QIAGEN). These PCR array kits analyze the expression levels of 84 AR signaling target genes (Table S1 in Supplementary Material) and 84 ER signaling target genes (Table S2 in Supplementary Material). Cbp/p300-interacting transactivator with Glu/Asp-rich carboxy-terminal domain 2 (Cited2, NM_053698), insulin-like growth factor binding protein 5 (Igfbp5, NM_012817), kallikrein B plasma 1 (Klkb1, NM_012725), and myelocytomatosis oncogene (Myc, NM_012603) were analyzed in both assays. Real-time PCR was performed using a StepOnePlus™ Real-Time PCR System (ThermoFisher SCIENTIFIC) with conditions of 95°C for 10 min, 40 cycles of 95°C for 15 s, and 60°C for 1 min, followed by a dissociation step according to the manufacturer’s instruction. Expression levels of AR signaling target genes were normalized by the mean expression levels of housekeeping genes (HKGs), actin beta (Actb, NM_031144), Hypoxanthine phosphoribosyltransferase 1 (Hprt1, NM_012583), lactate dehydrogenase A (Ldha, NM_017025), and ribosomal protein large P1 (Rplp1, NM_001007604) using the ΔΔCt method. Expression levels of ER signaling target genes were normalized by the mean expression levels of Hprt1 and Rplp1, which were selected from the HKGs by the software based on their consistent expression levels within all samples. Ct cut-off was set to 35 in both assays and treated as undetectable.

### Real-Time PCR

Five P3 male brains and five P35 male brains were collected under deep anesthesia by Z/K/X at 13.5 mg/90 μl/kg and stored at −80°C. Amygdala, hypothalamus, and telencephalon excluding amygdala and hippocampus were collected in a cryostat at −20°C. Tissues were homogenized in TRIzol™ Reagent (ThermoFisher SCIENTIFIC) and total RNA was extracted by chloroform. Total RNA was reverse transcribed by using High-Capacity cDNA Reverse Transcription Kit (ThermoFisher SCIENTIFIC). Expression levels of *Rattus norvegicus* Tmprss2 mRNA (NM_130424.3) and Foxa1 mRNA (NM_012742.1) were measured using Rplp1 mRNA (NM_001007604.2) as a reference HKG. Real-time PCR was performed using SensiFAST SYBR Master Mix (BioLine Reagent, London, United Kingdom) and primers (Tmprss2 forward: 5′-CACCTGCCATCCACATACAG-3′, reverse: 5′-CCAGAACTTCCAAAGCAAGC-3′; Foxa1 forward: 5′-GGAGGCCTACTCCTCTGTCC-3′, reverse: 5′-TTGGCGTAGGACATGTTGAA-3′; Rplp1 forward: 5′-GACGGTCACGGAGGATAAGA-3′, reverse: 5′-GCAGATGAGGCTTCCAATGT-3′). Real-time PCR was performed using a StepOnePlus™ Real-Time PCR System (ThermoFisher SCIENTIFIC) with conditions of 95°C for 2 min, 40 cycles of 95°C for 5 s, and 60°C for 30 s, followed by a dissociation step. The levels of each mRNA were normalized to Rplp1 mRNA using the ΔΔCt method.

### Immunohistochemistry and Fluorescent Microscopy

Immunohistochemistry was performed to investigate the location of Tmprss2 and Foxa1 protein in P3 male rat brains using rabbit monoclonal anti-TMPRSS2 antibody (EPR3861; abcam, Cambridge, United Kingdom) and anti-FOXA1 antibody (EPR10881: abcam). Briefly, five P3 male rats’ brains were collected in 4% paraformaldehyde (PFA) under deep anesthetized by Z/K/X at 13.5 mg/90 μl/kg. After 5 days in 4% PFA at 4°C, brains were soaked in 30% sucrose in 0.1 M phosphate buffer at 4°C until they sank. Brains were kept at −80°C until sectioning at 20 µm thickness on a cryostat at −20°C. Sections were incubated in 0.3% H_2_O_2_ in 20% methanol in 0.01 M phosphate buffered saline (PBS; pH 7.0) for 20 min to suppress endogenous peroxidase activity. Sections were then washed three times in PBS and incubated overnight at 4°C in the primary antibody at concentrations of 1:300 for anti-TMPRSS2 antibody and 1:200 for anti-FOXA1 antibody in blocking solution (0.5% Triton X, 2% normal goat serum in PBS). The next day, three subsequent washes in PBS were followed by incubation in biotinylated goat anti-rabbit IgG at 1:500 in blocking solution for 40 min. After the sections were washed in PBS three times, they were then incubated for 45 min in avidin–biotin complex (Vectastain Elite Kit, Vector, Burlingame, CA, USA) in blocking solution. The resulting complex was visualized by 3,3-diaminobenzidine after the sections were washed three times in PBS and rinsed in 0.05 M Tris–HCl buffer (pH 7.5). Immunohistochemistry without the primary antibodies served as control. The location of the immunoreactivities was identified by Nissl staining of the adjacent sections.

Number of Tmprss2-ir cells in the medial amygdala was counted in three 100 µm grids and averaged for each P3 male rat that were subcutaneously injected with BPA at 50 μg/kg/day (*n* = 5) or vehicle (*n* = 4) from P1 to P3. Number of Foxa1-ir cells in the hypothalamus was counted in three 100 µm grids and averaged for each P3 male rat that were subcutaneously injected with BPA at 50 μg/kg/day (*n* = 4) or vehicle (*n* = 4) from P1 to P3.

Co-localization of Tmprss2 or Foxa1 immunoreactivity and GnIH-EGFP was studied using a fluorescent microscope with bright field function. The secondary antibody (biotinylated goat anti-rabbit IgG) for immunohistochemistry was used at 1:200 to investigate the co-localization of Tmprss2 or Foxa1 and GnIH-EGFP.

### Statistics

Differential expression of AR and ER signaling target genes in the medial amygdala, number of Tmprss2-ir cells in the medial amygdala, and number of Foxa1-ir cells in the hypothalamus between BPA administered male rats and control rats were analyzed by Student’s *t*-test. Differential expression of Tmprss2 and Foxa1 in the amygdala, hypothalamus, and telencephalon excluding amygdala and hippocampus in P3 and P35 male rat brains was analyzed by one-way ANOVA followed by Fisher’s protected least significant difference test.

## Results

### Effect of BPA on AR and ER Signaling Target Gene Expression in the Medial Amygdala of Neonatal Male Rats

Differential expression of AR and ER signaling target genes in the medial amygdala by BPA administration was analyzed in neonatal male rats. Klkb1 (NM_012725), orosomucoid 1 (Orm1, NM_053288), and progastricsin (pepsinogen C) (Pgc, NM_133284) were undetectable within the 84 AR signaling target genes, and cytochrome P450 family 1 subfamily a polypeptide 1 (Cyp1a1, NM_012540), Klkb1 (NM_012725), nuclear receptor subfamily 0 group B member 2 (Nr0b2, NM_057133) were undetectable within the 84 ER signaling target genes (Table 1). Genes analyzed in both assays (Cited2, Igfbp5, Klkb1, Myc) produced similar results in terms of detectability, fold changes by BPA treatment, and *P* values by Student’s *t*-test (Table 1).

**Table 1:** Effect of Bisphenol A (BPA) on androgen receptor (AR) and estrogen receptor (ER) signaling target gene expression in the medial amygdala of neonatal male rats.

AR signaling target genes			ER signaling target genes		
Gene symbol	Fold changes	*P* value	Gene symbol	Fold changes	*P* value
Abcc4	1.10	0.627	Adora1	1.09	0.735
Abhd2	1.12	0.508	Ahr	1.11	0.837
Acsl3	0.99	0.913	Akap1	1.23	0.424
Adamts1	0.91	0.751	Apbb1	1.11	0.665
Aldh1a3	0.89	0.646	Bcar1	1.13	0.634
Appbp2	0.99	0.944	Bcl2l1	1.08	0.634
Ar	0.95	0.434	Bdnf	1.07	0.810
Atad2	1.13	0.655	Bmp4	0.89	0.673
Camkk2	1.14	0.191	Bmp7	6.46	0.585
Cenpn	0.98	0.948	Brca1	0.62	0.0541
Cited2	1.11	0.696	C3	1.00	0.925
Ackr3	1.01	0.929	Cav1	1.06	0.719
Cyp2u1	1.00	0.897	Ccl12	0.87	0.916
Dhcr24	1.21	0.387	Ccnd1	0.98	0.944
Eaf2	0.56	0.0645	Cited2	1.26	0.526
Elk1	1.14	0.391	Ckb	0.97	0.853
Ell2	1.01	0.935	Ctgf	0.76	0.359
Ern1	1.21	0.392	Ctsd	1.06	0.899
Errfi1	1.16	0.564	Cyp19a1	1.01	0.975
Fam105a	0.99	0.832	Cyp1a1	–	–
Fkbp5	1.08	0.571	Ebag9	15.05	0.266
Fos	0.80	0.252	Efna5	1.27	0.257
Fzd5	0.99	0.795	Egr3	0.88	0.651
Gucy1a3	1.13	0.222	Erbb2	1.19	0.521
Herc3	1.01	0.974	Erbb3	1.52	0.992
Hpgd	1.17	0.482	Esr1	1.21	0.515
Igf1r	1.10	0.564	Esr2	0.86	0.606
Igfbp5	0.96	0.753	Fos	0.79	0.351
Irs2	1.08	0.733	Foxa1	0.32	0.0147*
Jun	1.08	0.639	Fst	0.97	0.814
Klk1c2	2.43	0.135	G6pd	1.11	0.599
Klk4	0.95	0.994	Gper1 G	1.06	0.791
Klkb1	–	–	Hsp90aa1	0.96	0.731
Krt8	1.06	0.931	Igf1	0.86	0.503
Lama1	0.90	0.304	Igfbp4	1.51	0.164
Lifr	0.90	0.545	Igfbp5	0.98	0.867
Lrig1	1.00	0.975	Irs1	1.12	0.629
Lrrfip2	1.03	0.854	Junb	0.99	0.790
Maf	1.09	0.692	Klkb1	–	–
Map7d1	1.01	0.926	L1cam	1.02	0.994
Mme	1.00	0.957	Lgals1	1.01	0.883
Mt2A	1.15	0.563	Lpl	1.02	0.890
Myc	1.23	0.286	Ltbp1	0.89	0.549
Ncapd3	1.00	0.990	Maff	0.71	0.473
Ndrg1	1.27	0.127	Med1	1.07	0.844
Nfkb1	1.05	0.866	Mmp9	2.81	0.357
Nfkb2	0.95	0.816	Mta1	1.06	0.678
Nfkbia	1.01	0.773	Myc	1.33	0.348
Nkx3-1	0.64	0.102	Nab2	1.28	0.685
Orm1	–	–	Ncoa2	1.01	0.979
Pak1ip1	1.11	0.0870	Ncoa3	1.01	0.969
Pgc	–	–	Ncor1	0.81	0.285
Pias1	1.10	0.789	Ncor2	0.98	0.888
Pik3r3	1.07	0.794	Nov	0.89	0.450
Pmepa1	1.14	0.489	Nr0b1	0.84	0.534
Ppap2a	0.92	0.341	Nr0b2	–	–
Rab4a	0.99	0.901	Nr2f6	1.07	0.787
Rel	0.80	0.131	Nr3c1	0.93	0.775
Rela	0.97	0.788	Nr5a2	1.33	0.703
Ripk4	1.01	0.888	Nrip1	1.21	0.467
Sgk1	1.18	0.369	Nrp1	1.39	0.285
Slc26a2	1.04	0.801	Pdzk1	1.02	0.987
Slc45a3	0.75	0.509	Pelp1	0.95	0.650
Sms	0.98	0.685	Pgr	0.71	0.397
Snai2	0.79	0.328	Phb2	1.16	0.178
Sord	0.97	0.896	Ptch1	1.14	0.725
Sp1	1.06	0.692	Ptgs2	1.43	0.504
Spdef	1.15	0.475	Rala	1.06	0.587
Srf	1.12	0.564	Rara	1.27	0.548
Steap4	0.61	0.302	S100a6	0.87	0.539
Stk39	0.93	0.560	Safb	1.05	0.831
Tbc1d8	1.01	0.876	Snai1	1.15	0.725
Tiparp	1.56	0.988	Socs3	1.04	0.885
Tmprss2	2.17	0.0449*	Spp1	0.74	0.650
Tpd52	1.03	0.862	Tff1	–	–
Trib1	1.09	0.715	Tgfa	0.99	0.993
Tsc22d1	1.03	0.370	Tgfb3	1.16	0.466
Tsc22d3	1.12	0.587	Thbs1	1.05	0.861
Vapa	0.99	0.942	Vdr	1.15	0.686
Vipr1	0.96	0.821	Vegfa	1.14	0.719
Wipi1	1.08	0.655	Wisp2	1.01	0.974
Zbtb10	1.10	0.305	Wnt4	0.99	0.997
Zbtb16	0.99	0.889	Wnt5a	1.04	0.804
Zfp189	1.02	0.925	Xbp1	1.07	0.808

*Fold changes: ratio of the average of BPA group (*n* = 3, two siblings in one sample) to control group (*n* = 3, two siblings in one sample)*.

*–, under detectable*.

*P < 0.05 by Student’s t-test.

**Figure 1:**
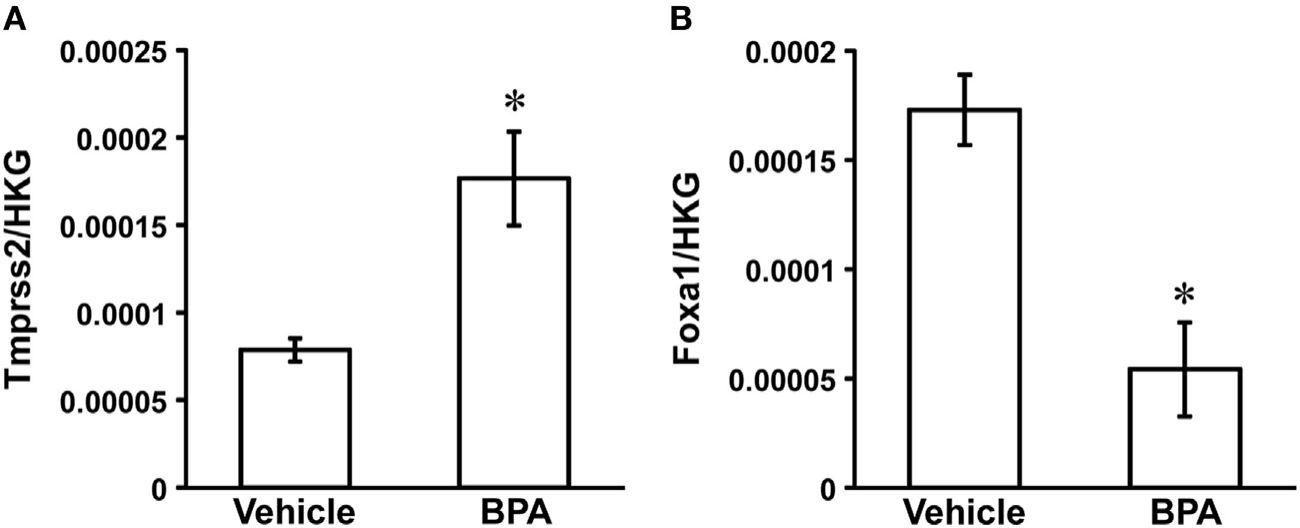
Effect of Bisphenol A (BPA) administration on Tmprss2 and Foxa1 gene expression in the neonatal male rat medial amygdala. **(A)** Effect of BPA administration on the ratio of Tmprss2 gene expression level relative to housekeeping gene (HKG). Each column and the vertical line represent the mean ± SEM (*n* = 3 samples, both sides of medial amygdala of two male siblings in one sample). *P < 0.05, vehicle vs. BPA by Student’s *t*-test. **(B)** Effect of BPA administration on the ratio of Foxa1 gene expression level relative to HKG. Each column and the vertical line represent the mean ± SEM (*n* = 3 samples, both sides of medial amygdala of two male siblings in one sample). *P < 0.05, vehicle vs. BPA by Student’s *t*-test.

An AR signaling target gene, Tmprss2 (NM_130424) was the only gene that was significantly increased in the medial amygdala of neonatal male rats by BPA treatment (fold change 2.17, *P* = 0.0449; Table 1; Figure 1A). An ER signaling target gene, Foxa1 (NM_012742) was the only gene that decreased significantly in the medial amygdala of neonatal male rats by BPA treatment (fold change 0.32, *P* = 0.0147; Table 1; Figure 1B).

### Distribution of Tmprss2 and Foxa1 Immunoreactive Cell Bodies in Neonatal Male Rat Brain

**Figure 2:**
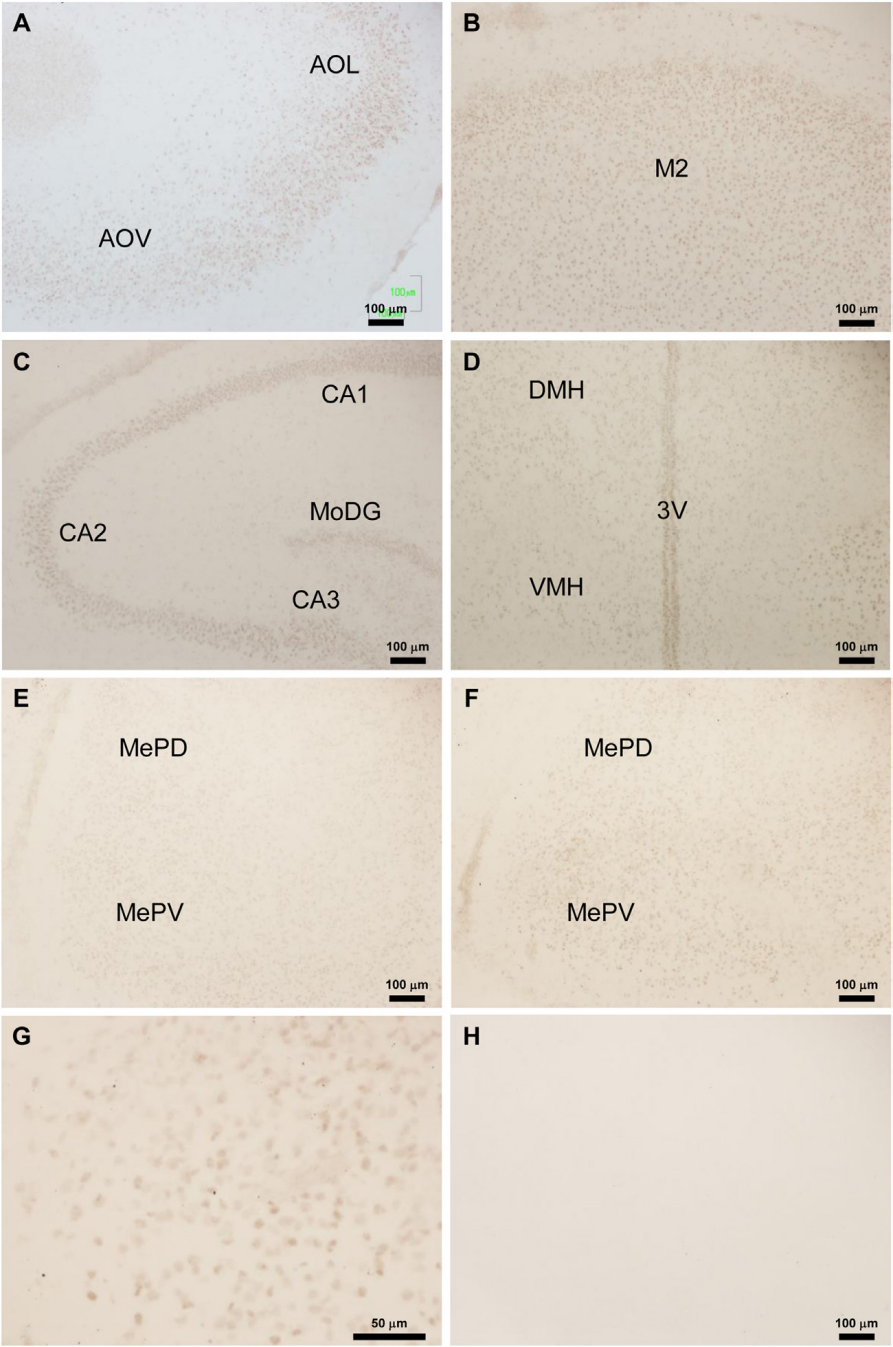
Location of Tmprss2-immunoreactive cells in the neonatal male rat brain. **(A)** Tmprss2-immunoreactive (ir) cells in the olfactory bulb of Bisphenol A (BPA) treated P3 rat. AOL, anterior olfactory nucleus, lateral part. AOV, anterior olfactory nucleus, ventral part. **(B)** Tmprss2-ir cells in the secondary motor cortex (M2) of BPA treated P3 rat. **(C)** Tmprss2-ir cells in the hippocampus of BPA treated P3 rat. CA1, field CA1 of the hippocampus; CA2, field CA2 of the hippocampus; CA3, field CA3 of the hippocampus; MoDG, molecular layer of the dentate gyrus. **(D)** Tmprss2-ir cells in the hypothalamus of BPA treated P3 rat. DMH, dorsomedial hypothalamic area; VMH, ventromedial hypothalamic area; 3V, third ventricle. **(E)** Tmprss2-ir cells in the amygdala of vehicle treated P3 rat. MePD, medial amygdaloid nucleus, posterodorsal part; MePV, medial amygdaloid nucleus, posteroventral part. **(F)** Tmprss2-ir cells in the amygdala of BPA treated P3 rat. **(G)** Higher magnification of MePV of BPA treated P3 rat **(F)**. **(H)** Immunohistochemistry without the first antibody showed no immunoreactive cellular structure.

Location of Tmprss2 and Foxa1-ir cell bodies in P3 male rat brains was analyzed by immunohistochemistry. Abundant Tmprss2-ir cell bodies were found in the olfactory bulb (Figure 2A), cerebral cortex (Figure 2B), hippocampus (Figure 2C), hypothalamus (Figure 2D), and amygdala (Figures 2E–G). Spindle-like Tmprss2-ir cell bodies of about 10 µm in diameter (Figure 2G) were consistently found in all brain areas. The weak blur staining of Tmprss2-ir cell bodies (Figure 2G) suggested that Tmprss2 exists in neuronal cellular membrane. Immunohistochemistry without the first antibody served as control (Figure 2H).

**Figure 3:**
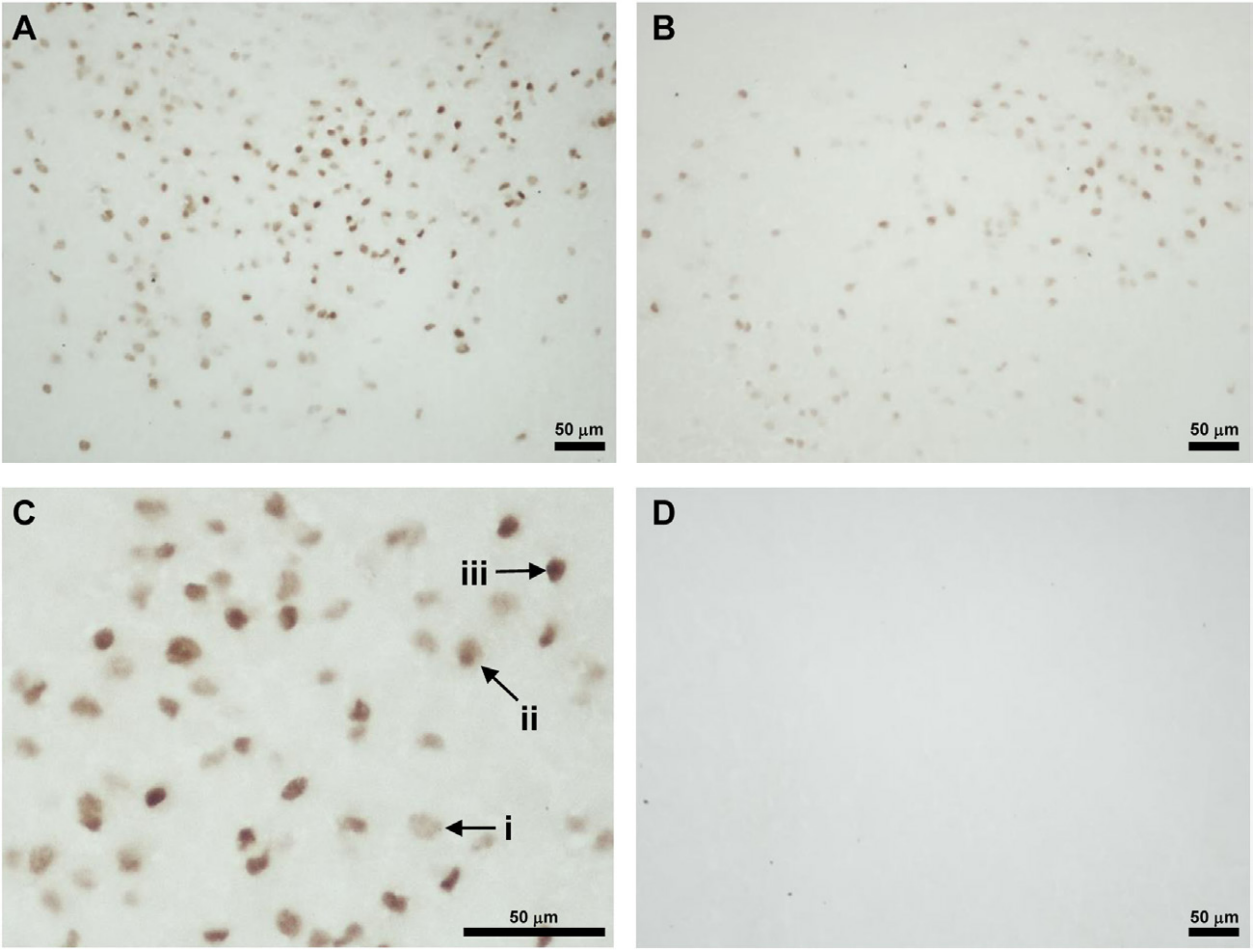
Location of Foxa1-immunoreactive cells in the neonatal male rat brain. **(A)** Foxa1-ir cells in the peduncular part of lateral hypothalamus (PLH) of vehicle treated P3 rat. **(B)** Foxa1-ir cells in the PLH of Bisphenol A treated P3 rat. **(C)** Higher magnification of Foxa1-ir cells in the PLH of vehicle treated P3 rat **(A)**. (i) A cell showing even staining in the cytoplasm and the nucleus. (ii) A cell showing darker staining in the nucleus than the cytoplasm. (iii) A cell showing intense staining only in the nucleus. **(D)** Immunohistochemistry without the first antibody showed no immunoreactive cellular structure.

Foxa1-ir cell bodies were only found in the peduncular part of lateral hypothalamus (PLH) (Figures 3A–C). Three kinds of staining were observed. The first type of staining was even through the cytoplasm and the nucleus, and the cellular diameter was about 10 µm (Figure 3C, i). The second type of staining was a weak staining in the cytoplasm and darker staining in the nucleus of about 7 µm in diameter (Figure 3C, ii). The third type of staining was an intense staining only in the nucleus (Figure 3C, iii). Immunohistochemistry without the first antibody served as control (Figure 3D).

### Effect of BPA Administration on the Number of Tmprss2-ir Cells in the Medial Amygdala and Foxa1-ir Cells in the Hypothalamus

**Figure 4:**
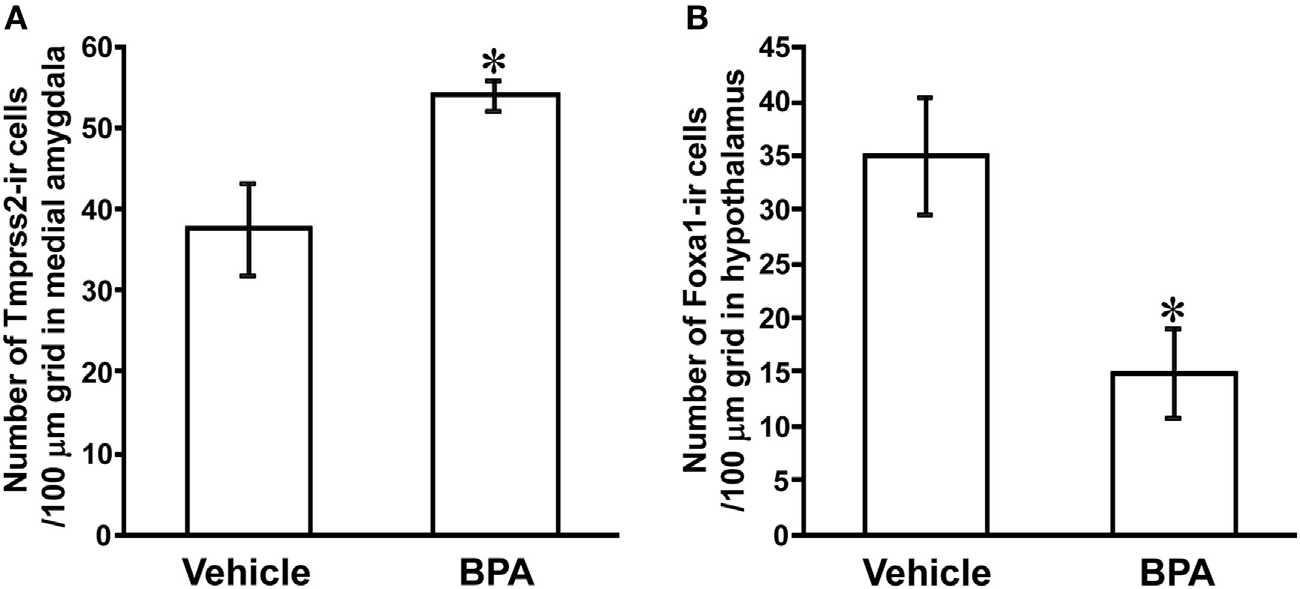
Effect of Bisphenol A (BPA) administration on the number of Tmprss2-ir cells in the medial amygdala and Foxa1-ir cells in the hypothalamus of neonatal male rat. **(A)** Effect of BPA administration on the number of Tmprss2-ir cells in 100 µm grid in the medial amygdala. Each column and the vertical line represent the mean ± SEM. *P < 0.05, vehicle (*n* = 4) vs. BPA (*n* = 5) by Student’s *t*-test. **(B)** Effect of BPA administration on the number of Foxa1-ir cells in 100 µm grid in the hypothalamus. Each column and the vertical line represent the mean ± SEM. *P < 0.05, vehicle (*n* = 4) vs. BPA (*n* = 4) by Student’s *t*-test.

To investigate the effect of BPA on Tmprss2 and Foxa1 protein expressions, number of Tmprss2-ir cells and Foxa1-ir cells were counted in the medial amygdala and hypothalamus, respectively, of P3 male rats that were daily administered with BPA or vehicle from P1 to P3. The number of Tmprss2-ir cells in the medial amygdala was significantly increased by BPA administration (*P* = 0.032; Figures 2E,F and 4A). On the other hand, the number of Foxa1-ir cells in the hypothalamus was significantly decreased by BPA administration (*P* = 0.042; Figures 3A,B and 4B).

### Co-Localization of Tmprss2 andEGFP-GnIH Cells in the Hypothalamus

**Figure 5:**
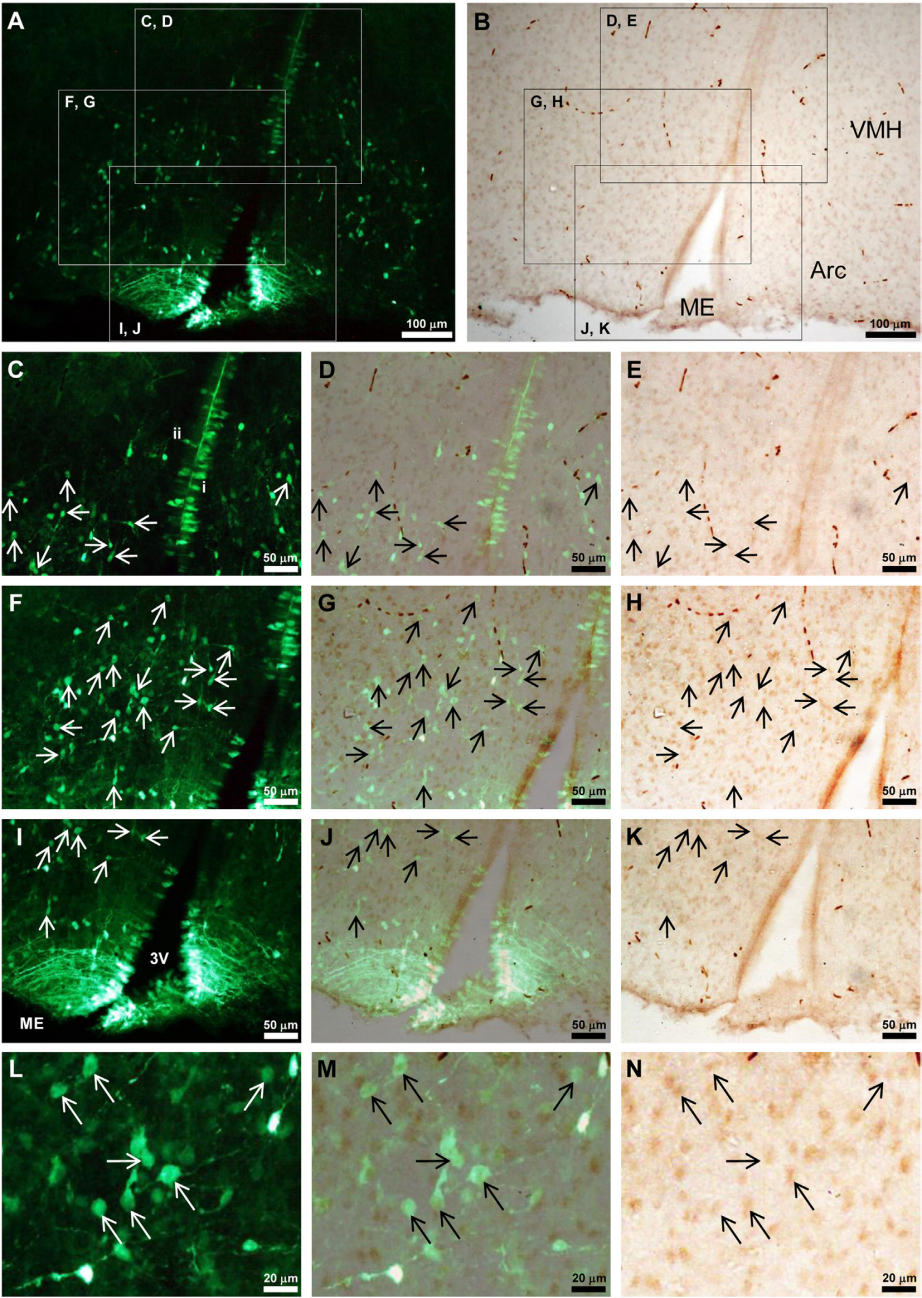
Tmprss2-immunoreactive EGFP-GnIH neurons in the hypothalamus of neonatal male rat. **(A,B)** EGFP-GnIH neurons **(A)** and Tmprss2-immunoreactive (ir) cells **(B)** in the identical field and focus of hypothalamus. VMH, ventromedial hypothalamic area; Arc, arcuate hypothalamic nucleus; ME, median eminence. **(C–E)** Higher magnification of highlighted area in panel **(A,B)** with identical focus. Panel **(D)** is the merged image of **(C,E)**. Arrows show Tmprss2-ir EGFP-GnIH neurons. [**(C)** i] An example of ventricle contacting rod-like EGFP-GnIH cell. [**(C)** ii] An example of EGFP-GnIH cell projecting its process to the ventricle. **(F–H)** Higher magnification of highlighted area in panel **(A,B)** with identical focus. Panel **(G)** is the merged image of **(F,H)**. Arrows show Tmprss2-ir EGFP-GnIH neurons. **(I–K)** Higher magnification of highlighted area in panel **(A,B)** with identical focus. Panel **(J)** is the merged image of **(I,K)**. Arrows show Tmprss2-ir EGFP-GnIH neurons. 3V, third ventricle. **(L–N)** Higher magnification of the central area in panel **(F–H)** with identical focus. Arrows show Tmprss2-ir EGFP-GnIH neurons.

Because Tmprss2 was highly expressed in the hypothalamus of P3 male rat (Figure 2D), where GnIH neuronal cell bodies are located, we investigated if some Tmprss2-ir cells are also EGFP-GnIH positive (17). Abundant EGFP-GnIH cells were observed from the ventral region of ventromedial hypothalamic nucleus (VMH) to the dorsal region of the arcuate nucleus (Arc) as well as along the third ventricle in P3 male rat hypothalamus (Figures 5A,C,F,I,L). The diameter of EGFP-GnIH cell bodies in the VMH and Arc was 6–11 µm and had typical GnIH neuronal structure with dendrite or axon-like processes (Figures 5A,F,L). 26.5 ± 6.8 (mean ± SD from four different brains) percent of EGFP-GnIH cells in the VMH and Arc were immunoreactive to Tmprss2 antibody (Figures 5A–N).

EGFP-GnIH cells along the third ventricle were different between the dorsal population (Figure 5C) and the ventral population in the median eminence (ME) (Figure 5I). The dorsal population of EGFP-GnIH cells along the third ventricle had two cell types. The first type had a rod-like structure with a diameter of 4–7 µm and they were directly attached to the ventricle (Figure 5C,i). The second type was round shape with a diameter of 4–7 µm situated in the sub-ventricular zone and had a protrusion to the ventricle (Figure 5C,ii). The ventral population of EGFP-GnIH cells in the ME had an oval shape with a minor axis of 4–7 µm directly contacted the ventricle and sent long processes to the external zone of the ME (Figure 5I). No clear cellular co-localization of Tmprss2 immunoreactivity was observed in EGFP-GnIH cells along the third ventricle either in the dorsal population (Figures 5A–E) or ventral population in the ME (Figures 5I–K).

### Developmental Change in Tmprss2 and Foxa1 Gene Expression in the Amygdala, Hypothalamus, and Telencephalon in the Male Rat Brain

**Figure 6:**
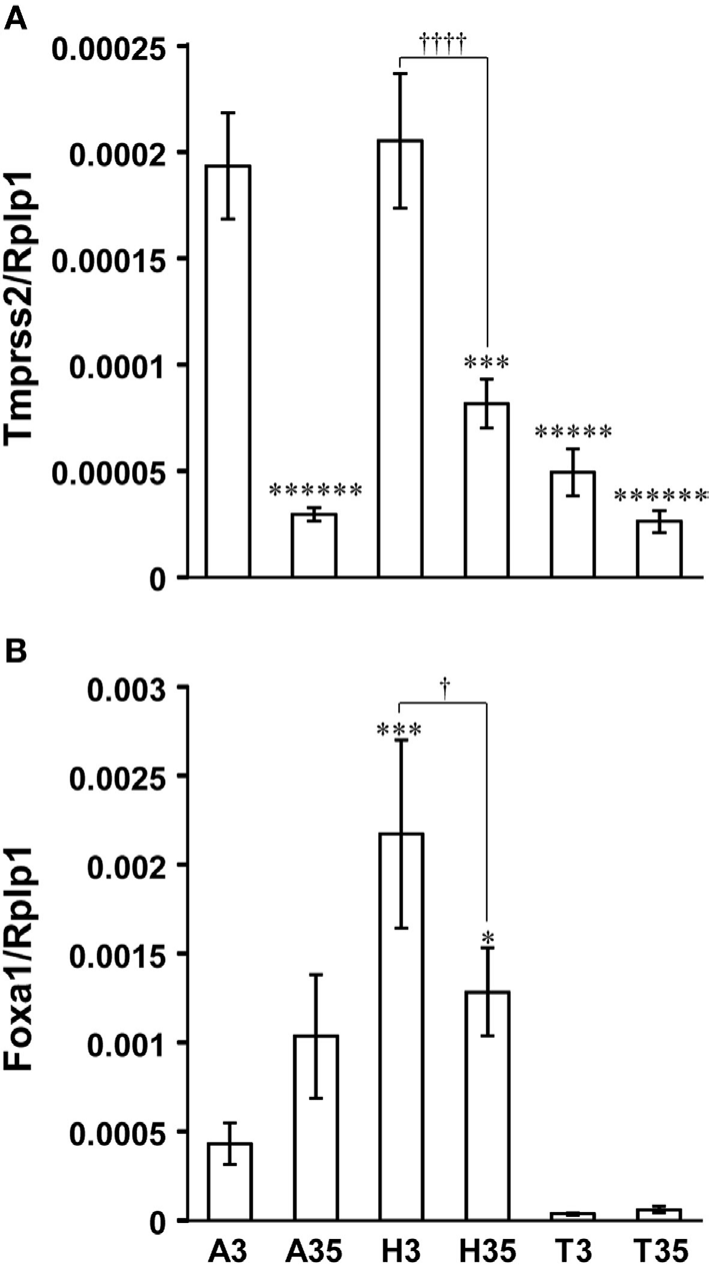
Developmental changes in Tmprss2 and Foxa1 gene expression in the amygdala, hypothalamus, and telencephalon excluding amygdala and hippocampus of male rat. **(A)** The ratio of Tmprss2 gene expression level relative to a housekeeping gene, Rplp1. Each column and the vertical line represent the mean ± SEM (*n* = 5 samples, one sample from one rat). ******P < 0.000001; *****P < 0.00001, ***P < 0.001 vs. amygdala of P3 male rat (A3) by one-way ANOVA followed by Fisher’s protected least significant difference (PLSD) test. ††††*P* < 0.0001, hypothalamus of P3 male rat (H3) vs. hypothalamus of P35 male rat (H35) by Student’s *t*-test. A35, amygdala of P35 male rat; T3, telencephalon excluding amygdala and hippocampus of P3 male rat; T35, telencephalon excluding amygdala and hippocampus of P35 male rat. **(B)** The ratio of Foxa1 gene expression level relative to Rplp1. Each column and the vertical line represent the mean ± SEM (*n* = 5 samples, one sample from one rat). ***P < 0.001; *P < 0.05 vs. A3 by one-way ANOVA followed by Fisher’s PLSD test. †*P* < 0.05, H3 vs. H35 by Student’s *t*-test.

Tmprss2 was equally highly expressed in the amygdala and hypothalamus compared with the telencephalon excluding amygdala and hippocampus in P3 male rat brain (Figure 6A). However, Tmprss2 expression in the amygdala and hypothalamus decreased significantly in P35 male rat equivalent to Tmprss2 expression levels in the telencephalon (Figure 6A).

Foxa1 was expressed significantly higher in the hypothalamus compared with the amygdala of P3 male rats (Figure 6B). Although Foxa1 expression in the hypothalamus decreased significantly in P35, it was significantly higher than its expression level in the amygdala of P3 male rats (Figure 6B). Foxa1 expression in the telencephalon excluding amygdala and hippocampus was almost under detectable level (Figure 6B).

## Discussion

To understand how perinatal exposure of BPA can modify the neurodevelopmental process and behavior, we first searched for BPA responsive genes from AR and ER signaling target genes by PCR array in the neonatal male rat medial amygdala that were subcutaneously administered with a reference dose of BPA (50 µg/kg BW/day) determined by United States EPA. We found upregulation of *Tmprss2*, an AR signaling target gene, and downregulation of *Foxa1*, an ER signaling target gene. Number of Tmprss2-ir cells in the medial amygdala was also increased and Foxa1-ir cells in the hypothalamus was also decreased in the hypothalamus by BPA administration to neonatal male rats.

*Tmprss2* encodes a multimeric protein that is translated into an N-terminal short cytoplasmic domain, a transmembrane domain, extracellular low-density lipoprotein receptor A domain, a scavenger receptor cysteine-rich domain, and a serine protease domain at the C-terminal (23). In human tissue, *TMPRSS2* is highly expressed in the small intestine, followed by heart, lung, liver, and small amounts in thymus and prostate. *TMPRSS2* is very highly expressed in the fetal brain but its expression level in the adult brain is minimum (23). *TMPRSS2* was found to be induced by androgen exposure to prostate cancer cells by microarray containing 1,500 cDNAs (24). It was shown that TMPRSS2 mediates androgen-induced prostate cancer cell invasion, tumor growth, and metastasis by stimulating a proteolytic cascade (25).

There is no study investigating the function of Tmprss2 in the brain besides only one study showing its possibility to mediate cancer pain by acting on trigeminal neurons (26). Significant decrease of *Tmprss2* expression in the amygdala and hypothalamus at 1 month of age suggests that Tmprss2 is related to neurogenesis or neuronal differentiation. In humans, TMPRSS2 is highly expressed in the fetus brain but its expression decreases to undetectable level in the adult brain (23). Tmprss2-ir cells were consistently distributed in olfactory bulb, cerebral cortex, hippocampus, amygdala, and hypothalamus in 3 days old male rats. We attempted Tmprss2 immunohistochemistry in 1-month-old and adult rat brains but we could not observe positive staining possibly due to its low expression level. The site of Tmprss2 expression corresponds well with that of AR expression in the rat brain (27, 28), suggesting that Tmprss2 expression is regulated by androgen.

In 3-day-old male rat hypothalamus, Tmprss2 was expressed in 26.5% of EGFP-GnIH neurons in the hypothalamus. EGFP-GnIH neurons were clustered from the ventral region of VMH to the dorsal region of Arc at this age, which was similar to fetal rat brain (29), but unlike GnIH neuronal population in the dorsomedial hypothalamic area in the adult (17, 30, 31). Abundant EGFP-GnIH cells were also found along the third ventricle, but clear expression of Tmprss2 was not observed in this region. EGFP-GnIH cells along the third ventricle can be determined as tanicytes from their morphology. Recent lineage-tracing experiments have shown that tanicytes along the third ventricle are hypothalamic stem cells (32) and postnatally differentiate into neurons and astrocytes in the hypothalamus (33). These results suggest that Tmprs2 is involved in the construction of neural and glial cellular architecture as a transmembrane protease in neonatal rat brain. Previous study has found that daily subcutaneous injection of a reference dose of BPA (50 µg/kg BW/day) to female Wister rats whose GnRH neurons express EGFP from P0 to P3 decreases numbers of GnIH-ir cell bodies, fiber density, and contacts on GnRH neurons and advances puberty at 1 month of age (34).

*FOXA1* belongs to the FOXA family of transcriptional regulators (FOXA1, FOXA2, and FOXA3) that play pivotal roles in mammalian development (35). FOXA proteins serve as pioneering factors in the early sequence of the transcriptional regulatory program. FOXA induces nucleosomal rearrangement that facilitates binding of other transcriptional regulators such as sex steroid hormone nuclear receptors (36). FOXA1 serves as a pioneering factor that is required for ERα to bind estrogen-response-element after estrogen stimulation (37). FOXA1 also facilitates AR/chromatin interactions at the regulatory loci near androgen responsive genes (38). It was shown that BPA promotes epithelial mesenchymal transition of ER-negative breast cancer cells through downregulation of FOXA1 by PI3K/Akt activation (39). It is possible that BPA also downregulates Foxa1 by activating PI3K/Akt in the brain.

*Foxa1* was highly expressed in the hypothalamus of P3 male rat but significantly decreased in the 1-month-old rat brain, suggesting that Foxa1 is also involved in neuronal differentiation. It was shown that Foxa1 is one of the regulators of neuronal differentiation of midbrain dopamine cells (40). A recent study showed that ablation of Foxa1 causes impaired development of subthalamic nucleus in mice (41), a brain region that is next to PLH where intense Foxa1-ir cells were found in P3 male rats in this study. Foxa1 immunoreactivity in the nucleus of the cells in PLH where AR and ER are also expressed (27) suggests that Foxa1 is involved in transcriptional activities of AR and ER in PLH.

Finally, it was shown that BPA upregulates Tmprss2 and downregulates Foxa1 in the neonatal male rat brain. Location of Tmprss2 and Foxa1-ir cells as well as significant decrease in Tmprss2 and Foxa1 expression in 1-month-old male rat brain suggest that BPA disturbs the neurodevelopmental process and behavior by modifying the expression of Tmprss2 and Foxa1 in the brain.

## Ethics Statement

All procedures were approved by Monash University, Animal Ethics Committee (MARP/2016/037).

## Author Contributions

TU conceived the project. TU, SM, and TS performed the experiments. TU wrote and IP edited the paper.

## Conflict of Interest Statement

The authors declare that the research was conducted in the absence of any commercial or financial relationships that could be construed as a potential conflict of interest.
