# Supplementary material for: Identification of Transmembrane Protease Serine 2 and Forkhead Box A1 As the Potential Bisphenol A Responsive Genes in the Neonatal Male Rat Brain
Source: Front Endocrinol (Lausanne). 2018 Mar 28;9:139. doi: 10.3389/fendo.2018.00139 (PMC5882795; doi:10.3389/fendo.2018.00139)
Supplement: Supplementary file 1 [file table_1.docx]

**Supplementary Table 1 |** Rat androgen receptor signaling target genes.

| **NCBI ID** | **Symbol** | **Description** |
| --- | --- | --- |
| NM_133411 | Abcc4 | ATP-binding cassette, subfamily C (CFTR/MRP), member 4 |
| NM_001106275 | Abhd2 | Abhydrolase domain containing 2 |
| NM_057107 | Acsl3 | Acyl-CoA synthetase long-chain family member 3 |
| NM_024400 | Adamts1 | ADAM metallopeptidase with thrombospondin type 1 motif, 1 |
| NM_153300 | Aldh1a3 | Aldehyde dehydrogenase 1 family, member A3 |
| NM_001100969 | Appbp2 | Amyloid beta precursor protein (cytoplasmic tail) binding protein 2 |
| NM_012502 | Ar | Androgen receptor |
| NM_001134879 | Atad2 | ATPase family, AAA domain containing 2 |
| NM_031338 | Camkk2 | Calcium/calmodulin-dependent protein kinase kinase 2, beta |
| NM_001008366 | Cenpn | Centromere protein N |
| NM_053698 | Cited2 | Cbp/p300-interacting transactivator, with Glu/Asp-rich carboxy-terminal domain, 2 |
| NM_053352 | Ackr3 | Chemokine (C-X-C motif) receptor 7 |
| NM_001024779 | Cyp2u1 | Cytochrome P450, family 2, subfamily u, polypeptide 1 |
| NM_001080148 | Dhcr24 | 24-dehydrocholesterol reductase |
| NM_172047 | Eaf2 | ELL associated factor 2 |
| NM_001108059 | Elk1 | ELK1, member of ETS oncogene family |
| XM_001054852 | Ell2 | Elongation factor RNA polymerase II 2 |
| NM_001191926 | Ern1 | Endoplasmic reticulum to nucleus signaling 1 |
| NM_001014071 | Errfi1 | ERBB receptor feedback inhibitor 1 |
| NM_001037648 | Fam105a | Family with sequence similarity 105, member A |
| NM_001012174 | Fkbp5 | FK506 binding protein 5 |
| NM_022197 | Fos | FBJ osteosarcoma oncogene |
| NM_173838 | Fzd5 | Frizzled homolog 5 (Drosophila) |
| NM_017090 | Gucy1a3 | Guanylate cyclase 1, soluble, alpha 3 |
| NM_001108631 | Herc3 | Hect domain and RLD 3 |
| NM_024390 | Hpgd | Hydroxyprostaglandin dehydrogenase 15 (NAD) |
| NM_052807 | Igf1r | Insulin-like growth factor 1 receptor |
| NM_012817 | Igfbp5 | Insulin-like growth factor binding protein 5 |
| NM_001168633 | Irs2 | Insulin receptor substrate 2 |
| NM_021835 | Jun | Jun oncogene |
| NM_012677 | Klk1c2 | Kallikrein 1-related peptidase C2 |
| NM_001004101 | Klk4 | Kallikrein-related peptidase 4 |
| NM_012725 | Klkb1 | Kallikrein B, plasma 1 |
| NM_199370 | Krt8 | Keratin 8 |
| NM_001108237 | Lama1 | Laminin, alpha 1 |
| NM_031048 | Lifr | Leukemia inhibitory factor receptor alpha |
| XM_232237 | Lrig1 | Leucine-rich repeats and immunoglobulin-like domains 1 |
| NM_001024761 | Lrrfip2 | Leucine rich repeat (in FLII) interacting protein 2 |
| NM_019318 | Maf | V-maf musculoaponeurotic fibrosarcoma oncogene homolog (avian) |
| NM_001109438 | Map7d1 | MAP7 domain containing 1 |
| NM_012608 | Mme | Membrane metallo-endopeptidase |
| NM_001137564 | Mt2A | Metallothionein 2A |
| NM_012603 | Myc | Myelocytomatosis oncogene |
| NM_001034000 | Ncapd3 | Non-SMC condensin II complex, subunit D3 |
| NM_001011991 | Ndrg1 | N-myc downstream regulated 1 |
| NM_001276711 | Nfkb1 | Nuclear factor of kappa light polypeptide gene enhancer in B-cells 1 |
| NM_001008349 | Nfkb2 | Nuclear factor of kappa light polypeptide gene enhancer in B-cells 2, p49/p100 |
| NM_001105720 | Nfkbia | Nuclear factor of kappa light polypeptide gene enhancer in B-cells inhibitor, alpha |
| NM_001034144 | Nkx3-1 | NK3 homeobox 1 |
| NM_053288 | Orm1 | Orosomucoid 1 |
| NM_001037356 | Pak1ip1 | PAK1 interacting protein 1 |
| NM_133284 | Pgc | Progastricsin (pepsinogen C) |
| NM_001106829 | Pias1 | Protein inhibitor of activated STAT, 1 |
| NM_022213 | Pik3r3 | Phosphoinositide-3-kinase, regulatory subunit 3 (gamma) |
| NM_001107807 | Pmepa1 | Prostate transmembrane protein, androgen induced 1 |
| NM_022538 | Ppap2a | Phosphatidic acid phosphatase type 2A |
| NM_013019 | Rab4a | RAB4A, member RAS oncogene family |
| XM_006221869 | Rel | V-rel avian reticuloendotheliosis viral oncogene homolog |
| NM_199267 | Rela | V-rel reticuloendotheliosis viral oncogene homolog A (avian) |
| NM_001107103 | Ripk4 | Receptor-interacting serine-threonine kinase 4 |
| NM_019232 | Sgk1 | Serum/glucocorticoid regulated kinase 1 |
| NM_057127 | Slc26a2 | Solute carrier family 26 (sulfate transporter), member 2 |
| NM_001135868 | Slc45a3 | Solute carrier family 45, member 3 |
| NM_001033899 | Sms | Spermine synthase |
| NM_013035 | Snai2 | Snail homolog 2 (Drosophila) |
| NM_017052 | Sord | Sorbitol dehydrogenase |
| NM_012655 | Sp1 | Sp1 transcription factor |
| NM_001109530 | Spdef | SAM pointed domain containing ets transcription factor |
| NM_001109302 | Srf | Serum response factor (c-fos serum response element-binding transcription factor) |
| NM_001044265 | Steap4 | STEAP family member 4 |
| NM_019362 | Stk39 | Serine/threonine kinase 39, STE20/SPS1 homolog (yeast) |
| NM_001191067 | Tbc1d8 | TBC1 domain family, member 8 |
| NM_001107679 | Tiparp | TCDD-inducible poly(ADP-ribose) polymerase |
| NM_130424 | Tmprss2 | Transmembrane protease, serine 2 |
| NM_001106421 | Tpd52 | Tumor protein D52 |
| NM_023985 | Trib1 | Tribbles homolog 1 (Drosophila) |
| NM_001109912 | Tsc22d1 | TSC22 domain family, member 1 |
| NM_031345 | Tsc22d3 | TSC22 domain family, member 3 |
| NM_031631 | Vapa | VAMP (vesicle-associated membrane protein)-associated protein A |
| NM_012685 | Vipr1 | Vasoactive intestinal peptide receptor 1 |
| NM_001127297 | Wipi1 | WD repeat domain, phosphoinositide interacting 1 |
| NM_024489 | Zbtb10 | Zinc finger and BTB domain containing 10 |
| NM_001013181 | Zbtb16 | Zinc finger and BTB domain containing 16 |
| NM_001107930 | Zfp189 | Zinc finger protein 189 |
