# Supplementary material for: Identification of Transmembrane Protease Serine 2 and Forkhead Box A1 As the Potential Bisphenol A Responsive Genes in the Neonatal Male Rat Brain
Source: Front Endocrinol (Lausanne). 2018 Mar 28;9:139. doi: 10.3389/fendo.2018.00139 (PMC5882795; doi:10.3389/fendo.2018.00139)
Supplement: Supplementary file 2 [file table_2.docx]

**Supplementary Table 2 |** Rat estrogen receptor signaling target genes.

| **NCBI ID** | **Symbol** | **Description** |
| --- | --- | --- |
| NM_017155 | Adora1 | Adenosine A1 receptor |
| NM_013149 | Ahr | Aryl hydrocarbon receptor |
| NM_053665 | Akap1 | A kinase (PRKA) anchor protein 1 |
| NM_080478 | Apbb1 | Amyloid beta (A4) precursor protein-binding, family B, member 1 (Fe65) |
| NM_012931 | Bcar1 | Breast cancer anti-estrogen resistance 1 |
| NM_031535 | Bcl2l1 | Bcl2-like 1 |
| NM_012513 | Bdnf | Brain-derived neurotrophic factor |
| NM_012827 | Bmp4 | Bone morphogenetic protein 4 |
| NM_001191856 | Bmp7 | Bone morphogenetic protein 7 |
| NM_012514 | Brca1 | Breast cancer 1 |
| NM_016994 | C3 | Complement component 3 |
| NM_031556 | Cav1 | Caveolin 1, caveolae protein |
| NM_001105822 | Ccl12 | Chemokine (C-C motif) ligand 12 |
| NM_171992 | Ccnd1 | Cyclin D1 |
| NM_053698 | Cited2 | Cbp/p300-interacting transactivator, with Glu/Asp-rich carboxy-terminal domain, 2 |
| NM_012529 | Ckb | Creatine kinase, brain |
| NM_022266 | Ctgf | Connective tissue growth factor |
| NM_134334 | Ctsd | Cathepsin D |
| NM_017085 | Cyp19a1 | Cytochrome P450, family 19, subfamily a, polypeptide 1 |
| NM_012540 | Cyp1a1 | Cytochrome P450, family 1, subfamily a, polypeptide 1 |
| NM_001009665 | Ebag9 | Estrogen receptor binding site associated, antigen, 9 |
| NM_053903 | Efna5 | Ephrin A5 |
| NM_017086 | Egr3 | Early growth response 3 |
| NM_017003 | Erbb2 | V-erb-b2 erythroblastic leukemia viral oncogene homolog 2, neuro/glioblastoma derived oncogene homolog (avian) |
| NM_017218 | Erbb3 | V-erb-b2 erythroblastic leukemia viral oncogene homolog 3 (avian) |
| NM_012689 | Esr1 | Estrogen receptor 1 |
| NM_012754 | Esr2 | Estrogen receptor 2 (ER beta) |
| NM_022197 | Fos | FBJ osteosarcoma oncogene |
| NM_012742 | Foxa1 | Forkhead box A1 |
| NM_012561 | Fst | Follistatin |
| NM_017006 | G6pd | Glucose-6-phosphate dehydrogenase |
| NM_133573 | Gper1 G | protein-coupled estrogen receptor 1 |
| NM_175761 | Hsp90aa1 | Heat shock protein 90, alpha (cytosolic), class A member 1 |
| NM_178866 | Igf1 | Insulin-like growth factor 1 |
| NM_001004274 | Igfbp4 | Insulin-like growth factor binding protein 4 |
| NM_012817 | Igfbp5 | Insulin-like growth factor binding protein 5 |
| NM_012969 | Irs1 | Insulin receptor substrate 1 |
| NM_021836 | Junb | Jun B proto-oncogene |
| NM_012725 | Klkb1 | Kallikrein B, plasma 1 |
| NM_017345 | L1cam | L1 cell adhesion molecule |
| NM_019904 | Lgals1 | Lectin, galactoside-binding, soluble, 1 |
| NM_012598 | Lpl | Lipoprotein lipase |
| NM_021587 | Ltbp1 | Latent transforming growth factor beta binding protein 1 |
| NM_001130573 | Maff | V-maf musculoaponeurotic fibrosarcoma oncogene homolog F (avian) |
| NM_001134361 | Med1 | Mediator complex subunit 1 |
| NM_031055 | Mmp9 | Matrix metallopeptidase 9 |
| NM_022588 | Mta1 | Metastasis associated 1 |
| NM_012603 | Myc | Myelocytomatosis oncogene |
| NM_001134874 | Nab2 | Ngfi-A binding protein 2 |
| NM_031822 | Ncoa2 | Nuclear receptor coactivator 2 |
| XM_006224744 | Ncoa3 | Nuclear receptor coactivator 3 |
| NM_001271103 | Ncor1 | Nuclear receptor co-repressor 1 |
| NM_001108334 | Ncor2 | Nuclear receptor co-repressor 2 |
| NM_030868 | Nov | Nephroblastoma overexpressed gene |
| NM_053317 | Nr0b1 | Nuclear receptor subfamily 0, group B, member 1 |
| NM_057133 | Nr0b2 | Nuclear receptor subfamily 0, group B, member 2 |
| NM_139113 | Nr2f6 | Nuclear receptor subfamily 2, group F, member 6 |
| NM_012576 | Nr3c1 | Nuclear receptor subfamily 3, group C, member 1 |
| NM_021742 | Nr5a2 | Nuclear receptor subfamily 5, group A, member 2 |
| NM_001100560 | Nrip1 | Nuclear receptor interacting protein 1 |
| NM_145098 | Nrp1 | Neuropilin 1 |
| NM_031712 | Pdzk1 | PDZ domain containing 1 |
| NM_001024270 | Pelp1 | Proline, glutamate and leucine rich protein 1 |
| NM_022847 | Pgr | Progesterone receptor |
| NM_001013035 | Phb2 | Prohibitin 2 |
| NM_053566 | Ptch1 | Patched homolog 1 (Drosophila) |
| NM_017232 | Ptgs2 | Prostaglandin-endoperoxide synthase 2 |
| NM_031093 | Rala | V-ral simian leukemia viral oncogene homolog A (ras related) |
| NM_031528 | Rara | Retinoic acid receptor, alpha |
| NM_053485 | S100a6 | S100 calcium binding protein A6 |
| NM_022394 | Safb | Scaffold attachment factor B |
| NM_053805 | Snai1 | Snail homolog 1 (Drosophila) |
| NM_053565 | Socs3 | Suppressor of cytokine signaling 3 |
| NM_012881 | Spp1 | Secreted phosphoprotein 1 |
| NM_057129 | Tff1 | Trefoil factor 1 |
| NM_012671 | Tgfa | Transforming growth factor alpha |
| NM_013174 | Tgfb3 | Transforming growth factor, beta 3 |
| NM_001013062 | Thbs1 | Thrombospondin 1 |
| NM_017058 | Vdr | Vitamin D (1,25- dihydroxyvitamin D3) receptor |
| NM_031836 | Vegfa | Vascular endothelial growth factor A |
| NM_031590 | Wisp2 | WNT1 inducible signaling pathway protein 2 |
| NM_053402 | Wnt4 | Wingless-type MMTV integration site family, member 4 |
| NM_022631 | Wnt5a | Wingless-type MMTV integration site family, member 5A |
| NM_001004210 | Xbp1 | X-box binding protein 1 |
